# Distribution of *Anopheles* vectors and potential malaria transmission stability in Europe and the Mediterranean area under future climate change

**DOI:** 10.1186/s13071-018-3278-6

**Published:** 2019-01-08

**Authors:** Elke Hertig

**Affiliations:** Institute of Geography, University of Augsburg, Alter Postweg 118, 86135 Augsburg, Germany

**Keywords:** Vector distributions, Malaria epidemiology, Malaria reemergence, Regional Climate Models

## Abstract

**Background:**

In the scope of climate change the possible recurrence and/or expansion of vector-borne diseases poses a major concern. The occurrence of vector competent *Anopheles* species as well as favorable climatic conditions may lead to the re-emergence of autochthonous malaria in Europe and the Mediterranean area. However, high-resolution assessments of possible changes of *Anopheles* vector distributions and of potential malaria transmission stability in the European-Mediterranean area under changing climatic conditions during the course of the 21st century are not available yet.

**Methods:**

Boosted Regression Trees are applied to relate climate variables and land cover classes to vector occurrences. Changes in future vector distributions and potential malaria transmission stability due to climate change are assessed using state-of-the art regional climate model simulations.

**Results:**

Distinct changes in the distributions of the dominant vectors of human malaria are to be expected under climate change. In general, temperature and precipitation changes will lead to a northward spread of the occurrences of *Anopheles* vectors. Yet, for some Mediterranean areas, occurrence probabilities may decline.

**Conclusions:**

Potential malaria transmission stability is increased in areas where the climatic changes favor vector occurrences as well as significantly impact the vectorial capacity. As a result, vector stability shows the highest increases between historical and future periods for the southern and south-eastern European areas. *Anopheles atroparvus*, the dominant vector in large parts of Europe, might play an important role with respect to changes of the potential transmission stability.

**Electronic supplementary material:**

The online version of this article (10.1186/s13071-018-3278-6) contains supplementary material, which is available to authorized users.

## Background

In Europe and the Mediterranean area widespread elimination of malaria could be achieved during the 20th century [1]. The decline of malaria is strongly related to socio-economic improvements such as wealth, life expectancy and urbanization [2]. However, in recent years an increasing number of imported malaria cases occurs due to international travel and immigrants from malaria-endemic countries [3]. Together with the occurrence of vector competent *Anopheles* species and favorable climatic conditions autochthonous malaria cases may re-emerge in countries where malaria was previously eradicated. Since the late 1990s locally transmitted cases have been reported in Germany, the Netherlands, Spain, France, Italy, Greece and Turkey [4]. In general, malaria transmission in Europe is highly seasonal owing to temperate climatic conditions. The Mediterranean area, with mild and wet winters and hot and dry summers, has been and still is suitable for malaria transmission. The dominant *Anopheles* vector species in Europe and the Mediterranean are currently *Anopheles atroparvus*, *An*. *labranchiae*, *An*. *messeae*, *An*. *sacharovi*, *An*. *sergentii* and *An*. *superpictus* [5]. Autochthonous malaria in Europe is mainly caused by the human malaria parasite *Plasmodium vivax* [6].

Projections of future climate change show that there will be significant warming rates until the end of the 21st century, strongest over north-eastern Europe in winter and over Central Europe and the Mediterranean area in summer. Precipitation is projected to increase mainly over North Europe in winter, whereas South Europe and the Mediterranean will likely see decreases the whole year round [7]. An assessment of possible future changes of the length of the malaria transmission season using climate output from General Circulation Models (GCMs) and different malaria impact models shows that until the 2080s a northward shift of the malaria epidemic belt over central-northern Europe might occur [6]. Medlock & Leach [8] concluded in a review study for the UK that under climate change more than two months of *P. vivax* transmission by 2030, and four months by 2080 could arise. For Lower Saxony in Germany Schröder & Schmidt [9] showed that in the second half of the 20th century the temperature-dependent malaria transmission rate of *An. atroparvus* from May to October was two months and might take values up to five or six months until the end of the 21st century.

In the context of global climate change GCMs are typically used to obtain large-scale climate information for future periods. However, GCMs do not provide reliable information on regional to local scales. Thus, different downscaling approaches have been developed. In general, they can be divided into statistical downscaling (establishing a statistical link between large-scale atmospheric predictors and regional to local climate) and dynamical downscaling, i.e. nesting a regional climate model (RCM) into a GCM with higher grid box resolution [10]. Current RCMs can reproduce the most important climatic features such as temperature and precipitation at regional scales, but some important biases still remain. These refer for instance to local heavy precipitation events and are related to the convective parameterizations, grid resolution and other aspects of the model formulation [10]. Therefore, a correction method such as quantile mapping (QM) is usually applied to the RCM output [11]. QM adjusts the cumulative distribution function of a simulated variable to match the cumulative distribution function of observed values. Gutiérrez et al. [11] found in an intercomparison of different downscaling techniques for temperature and precipitation in the European area that empirical QM including a seasonal component performs with very small biases.

The present study investigates distribution changes of dominant *Anopheles* vectors of human malaria in the greater European area for the 21st century under climate change. It builds on previous work of Sinka et al. [5] and Kuhn et al. [12] who modelled the current distribution of dominant *Anopheles* vectors in Europe using environmental and climatic variables. Variables in these studies included i.a. monthly temperature and precipitation values, elevation, and land cover classes [12, 13]. The focus of the present study is to assess future vector distribution changes due to climate change using state-of-the art RCM simulations. As in Sinka et al. [5, 13] Boosted Regression Trees (BRTs, [14]) are applied to relate climate variables and land-cover classes to vector occurrences. Bias-corrected temperature and precipitation from two different GCM-RCM setups under two emission scenarios are used to assess regional climate suitability of *Anopheles* vectors in Europe and the Mediterranean area under future climate change. Only changes of the climatic influences on the vector distribution are considered in the regional projections, since environmental effects such as land use changes are currently not implemented in the standard RCM generation. Finally, potential malaria transmission stability during the 21st century is assessed, which builds on the vector stability index of [15].

## Methods

### Anopheles occurrence data

Occurrence data of the dominant *Anopheles* vectors in Europe come from the distribution maps of Sinka et al. [5]. These authors used the Malaria Atlas Project library, a literature research and expert opinion maps to produce maps of *Anopheles* occurrences. Occurrence data refer basically to the period 1985–2009. The presence-absence information of the six *Anopheles* vectors (*An. atroparvus*, *An*. *labranchiae*, *An*. *messeae*, *An*. *sacharovi*, *An*. *sergentii* and *An*. *superpictus*), which is provided as Shapefiles in the electronic supplementary material of Sinka et al. [5], was rasterized to a 0.25° resolution to match the grid resolution of the observation-based climate data.

### Climate data

#### Observational data

Mean, minimum and maximum temperature as well as precipitation data were taken from the daily 0.25° E-OBS dataset version 17 provided by the European Climate Assessment & Dataset (ECA&D [16]). A European-Mediterranean domain was selected, covering 75.375–25.375°N and 19.875°W49.875°E. Data in the time period 1950–2009 were selected. The time period 1985–2009, which is mainly used for subsequent analysis, was filtered for missing values for each month separately. A particular month was considered complete if there were less than three missing days per month, and the time series was considered complete if there were less than four missing months in the period 1985–2009. Grid boxes which did not meet these conditions were removed. Monthly temperature means (in Kelvin K) as well as monthly precipitation totals were calculated from the daily data.

#### Model data

RCM simulations carried out in the framework of EURO-CORDEX (European branch of the Coordinated Regional Climate Downscaling Experiment [17]) are employed. In the present study data with the grid resolution of about 50 km (0.44° on a rotated grid) are considered and conservatively regridded to 0.25° in order to match the E-OBS grid resolution. KNMI-RACMO22E driven by the GCM EC-EARTH as well as CLMcom-CCLM4-8-17 driven by MPI-ESM-LR were chosen as RCM simulations. The two RCMs were selected according to their good performance over Europe in the observational period [18]. Historical runs for the period 1950–2005 (KNMI-RACMO22E) and 1960–2005 (CLMcom-CCLM4-8-17) as well as scenario runs for the period 2006–2100 under RCP4.5 and RCP8.5 scenario assumptions [19] were available for subsequent analyses.

#### Land cover data

Land cover information was used in the form of 22 categories of land cover from the GlobCover project [20]. GlobCover3 2009 V.2.3 land cover map is derived by an automatic and regionally-tuned classification of a time series of global ERIS (Medium Resolution Imaging Spectrometer Instrument) Fine Resolution mosaics for the year 2009. The land cover classes, defined with the United Nations Land Cover Classification System, have a 300 × 300 m resolution. To match the E-OBS grid resolution, land cover information was calculated as percent coverage of each class in a particular 0.25° grid box.

### Correction of model data

Transfer functions were defined to match the RCM output of a variable *P*_*m*_ (temperature, precipitation) in the historical period with the statistical properties of a variable *P*_*o*_ from the E-OBS observations. The non-parametric empirical quantile method suggested in [21] and implemented in the *qmap* package in R was used. The transfer functions were subsequently used to correct the RCM output of the historical and the future periods. According to Gudmundsson et al. [21], the transformation is defined as:

1 $$ {P}_o={F}_o^{-1}\left({F}_m\left({P}_m\right)\right) $$

where *F*_*m*_ is the cumulative distribution function (CDF) of *P*_*m*_ and ${F}_o^{-1}$ is the inverse CDF (quantile function) corresponding to *P*_*o*_.

The empirical CDFs were approximated using empirical percentiles at a fixed interval of 0.01. Values in between the percentiles were approximated using a linear interpolation. A threshold for the correction of the number of wet days was estimated from the empirical probability of non-zero values in *P*_*o*_. The correction of the daily values at each grid box was done for each month separately to account for seasonality.

The performance of QM was assessed by using a split-sampling validation approach. For KNMI-RACMO22E the historical model output comprises the years 1950–2005, which was split into the 30-year calibration periods 1950–1979 and 1976–2005. Bias correction was done for each of the two calibration periods and the performance was validated in the two, from the correction independent, 26-year periods 1980–2005 and 1950–1975, respectively. For CLMcom-CCLM4-8-17 historical model output was available for the period 1960–2005 and the 25-year calibration periods 1960–1984 and 1981–2005 were used for setting up the transfer functions between observed and modelled values. The statistical bias correction was subsequently validated in the independent 21-year periods 1985–2005 and 1960–1980, respectively. Root mean square error (RMSE) between observed and modelled values was used as performance measure. RMSE is calculated for the arithmetic means across all grid cells, assuming an equal weight for each cell. Thus, it serves as a cumulative measure of the bias over the considered domain.

### Distribution modelling and projection

Climate data (mean, minimum and maximum temperature and precipitation) of each month separately in the time period 1985–2009 as well as the land cover data served as predictors for vector occurrences. In order to quantify the relationships between vector occurrences with climate and land cover variables and to map and project the occurrences under present and future climate conditions Boosted Regression Trees (BRTs) were used. Detailed descriptions of BRT are provided by Elith et al. [14] and Hastie et al. [22]. BRT combines regression trees and boosting. BRT attempts to minimize a loss function, which involves jointly optimizing the number of trees, learning rate, and tree complexity. The learning rate is used to shrink the contribution of each tree as it is added to the model. Slowing the learning rate increases the number of trees required. In general, a smaller learning rate (and consequently a larger number of trees) is preferable. Tree complexity (number of nodes in a tree) relates to the interaction order in the predictand. With increasing tree complexity, learning rate must be decreased if sufficient trees are to be fitted to minimize predictive error. The tree complexity should reflect the correct interaction order in the response variable. However, since an adequate tree complexity is usually unknown, it is best evaluated using independent data. As in Elith et al. [14] the optimal number of trees, learning rate and tree complexity were estimated with a cross-validation approach, using deviance reduction as performance measure. The *dismo* and *gbm* packages in R were used to assess the optimal number of boosting trees using 10-fold cross-validation. In the present study models were developed with 50% of the data, and were validated with the remaining data. Tree complexity of 2 up to 8, and learning rates of 0.005, 0.1 and 0.5 were evaluated.

The modeling of the vector distributions using BRT requires both presence and absence data. The lack of confirmed absences in the occurrence data was addressed by the production of artificial absence data, called pseudo-absences. The pseudo-absences are all grid boxes outside of the suitable area, which is estimated by a rectilinear surface range envelope [23]. Following the recommendation of Barbet-Massin et al. [24] the same number of pseudo-absences as presences was tested (ratio 1:1, with 1000 presences and 1000 pseudo-absences randomly selected from the available data). Additionally, ratios of 5:1 (5000 pseudo-absences and 1000 presences) and 10:1 (5000 pseudo-absences and 500 presences) were also tested, since Sinka et al. [5], although using different predictor data and BRT setup, found the best overall performance for the European and Middle Eastern *Anopheles* species with a ratio of 10:1 pseudo-absences to presence. Model validation was subsequently done using the remaining independent data not used for model building. The BRT model was used to predict vector occurrences to the independent data and the result was taken for evaluation of the model. As statistics on predictive performance deviance, correlation, discrimination and Kappa were estimated and results were also evaluated visually. Details on cross-validation and performance measures can be found for instance in [25–27].

Subsequently, the best performing BRT configuration was used to project vector occurrences under future climate change. For this purpose, the bias-corrected RCM data were taken as new predictor data in the BRTs. The projected occurrences were evaluated for the historical period 1985–2005, and the two scenario periods 2040–2060 and 2080–2100.

### Potential malaria transmission stability

The vector stability index (VSI) of Kiszewski et al. [15] is used to generate maps of the future potential malaria transmission stability under climate change:

2 $$ \boldsymbol{VSI}=\sum \limits_{\boldsymbol{m}=\mathbf{1}}^{\mathbf{12}}{\boldsymbol{a}}_{\boldsymbol{i},\boldsymbol{m}}^{\mathbf{2}}{\boldsymbol{p}}_{\boldsymbol{i},\boldsymbol{m}}^{\boldsymbol{E}}/-\mathbf{\ln}\left({\boldsymbol{p}}_{\boldsymbol{i},\boldsymbol{m}}\right) $$

where m is month; i is vector; a is the human-biting proportion (0−1); p is the daily survival rate (0−1); and E is the length of extrinsic incubation period in days (for *P. vivax* E = 105/T-14.5). VSI was calculated for each vector i. The parameters a and p for each *Anopheles* species were taken from the publication of Kiszewski et al. [15]. Within the calculation of the length of extrinsic incubation period E, mean temperature (T) of the historical time slice 1985–2005 as well as of the future time slices 2040–2060 and 2080–2100, taken from the bias-corrected RCM data, were used. The vector-specific VSI results were integrated into an overall information by multiplying the VSI value with the modelled occurrence probability for each vector at each grid box. The VSI of that vector which has the highest value of combined VSI and occurrence probability at a particular grid box was subsequently mapped.

## Results

### Vector and climate data

The filtering procedure (completeness check) of the climate data resulted in 20,632 grid boxes to be used for subsequent analyses. Grid boxes with vector presence but no available climate data were excluded from further analyses. The rasterization of the *Anopheles* occurrence data to the 0.25° grid yielded 7850 grid boxes of presences for *An. atroparvus* (38% of the European-Mediterranean land area with available climate data), 1494 grid boxes for *An. labranchiae* (7.2%), 13,490 grid boxes for *An. messeae* (65.4%), 2449 grid boxes *An. sacharovi* (11.9%), 1221 grid boxes for *An. sergentii* (5.9%) and 2495 grid boxes for *An. superpictus* (12.1%). The rasterized distribution maps are given in Additional file 1: Figure S1.

### Regional climate model bias correction

Correction of the RCM data using empirical quantile mapping reduced the bias of KNMI-RACMO22E precipitation by 0.17 mm/day to a bias, averaged across all months, of 0.43 mm/day. For temperature, the bias was reduced by 1.35 K to an average bias of 1.14 K for mean temperature, by 1.62 K to 1.2 K for minimum temperature, and by 1.66 K to 1.14 K for maximum temperature. The bias of CLMcom-CCLM4-8-17 precipitation was0.38 mm/day lower compared to the raw RCM output and amounted after bias correction to 0.47 mm/day averaged over all months. Mean temperature bias was reduced by 0.62 K to 0.8 K, minimum temperature by 0.52 K to 0.84 K, and maximum temperature by 1.56 K to 0.87 K. The individual monthly values for all variables of both RCMs are tabulated in Additional file 1: Table S1.

### Vector distribution models

From the different configurations tested the best performance of the BRTs was achieved with learning rate = 0.005 and bag fraction = 0.5. For *An. atroparvus* and *An. messeae*, which have a widespread occurrence in the European area and thus have a more equal number of grid boxes with presence to absence, the ratio 1:1 of pseudo-absence to presence data and tree complexity of 2 yields the best BRT model performance. For *An*. *labranchiae*, *An. sacharovi*, *An. sergentii* and *An*. *superpictus*, which have a smaller geographical range, the ratio 10:1 of pseudo-absence to presence and tree complexity of 3 gave the best results. Additional file 1: Table S2 and Figure S2 show for each vector the predictive performance of the best-performing BRT and maps of the modelled occurrence probabilities, respectively. The modelled spatial distributions showed a high agreement with the reference occurrence data (Additional file 1: Figure S1). The most noticeable discrepancy occurred for *An. atroparvus* over the southern parts of the Iberian Peninsula, where vector presence is missed in the modelling.

The information about the contribution of each predictor variable to the model is based on the number of times a variable is selected for splitting, weighted by the squared improvement to the model as a result of each split, and averaged over all trees [14]. For *An. atroparvus* the most important temperature predictors (relative importance > 5%) were temperature conditions in early spring. The fitted functions revealed that the 0 °C threshold is of particular importance in this regard. A similar relationship emerged for *An*. *labranchiae*. For *An*. *sacharovi* and *An*. *superpictus*, maximum temperatures in early spring up to approx. 10–15 °C were positively related to occurrence. For *An*. *messeae*, mean temperature in spring was selected in the BRTs. These results point to the relevance of temperature conditions at the end of the hibernation period/beginning of the active period. For *An*. *superpictus*, high maximum temperatures in autumn were favorable as well. For *An*. *messeae*, an optimum maximum temperature range in autumn at the beginning of the hibernation period was also relevant. Overall, vector occurrences showed the highest dependence on temperature conditions in the transitional seasons at the beginning/end of the active and hibernation periods.

The contribution of precipitation in summer and early autumn in the BRTs for *An*. *atroparvus* indicates the importance of sufficient water during the aquatic life-cycle. For *An*. *messeae* precipitation in spring and early summer was highly relevant; for *An*. *labranchiae* precipitation in spring and early autumn played an important role. *Anopheles sacharovi* showed a great dependence on climate conditions (minimum and mean temperature, rainfall) in summer, the peak time of the adult activity. The occurrence of the North African species *An. sergentii* was almost completely governed by rainfall conditions in summer. While in general precipitation was positively connected with *Anopheles* occurrences, very high monthly precipitation amounts can also have a reverse impact on occurrences. This applies to spring precipitation amounts for *An. messeae*, and to summer precipitation for *An. sacharovi* and *An*. *sergentii*.

Land cover classes with a relative predictor importance > 5% were only present in the BRTs for *An. atroparvus*, *An. labranchiae* and *An. superpictus*. Rainfed croplands (high fractions of this land cover class were positively related to *An. atroparvus* occurrence; closed to open (> 15%) shrubland were negatively related to the occurrence of the Mediterranean species *An. labranchiae* and *An. superpictus*; mosaic cropland (50–70%)/vegetation (20–50%) were positively related to *An. labranchiae* occurrence; and closed (> 40%) broadleaved deciduous forest were positively related to *An. superpictus* occurrence. In summary, climatic predictors clearly dominated as important predictors in the BRTs.

### Projections of vector distributions

**Fig. 1:**
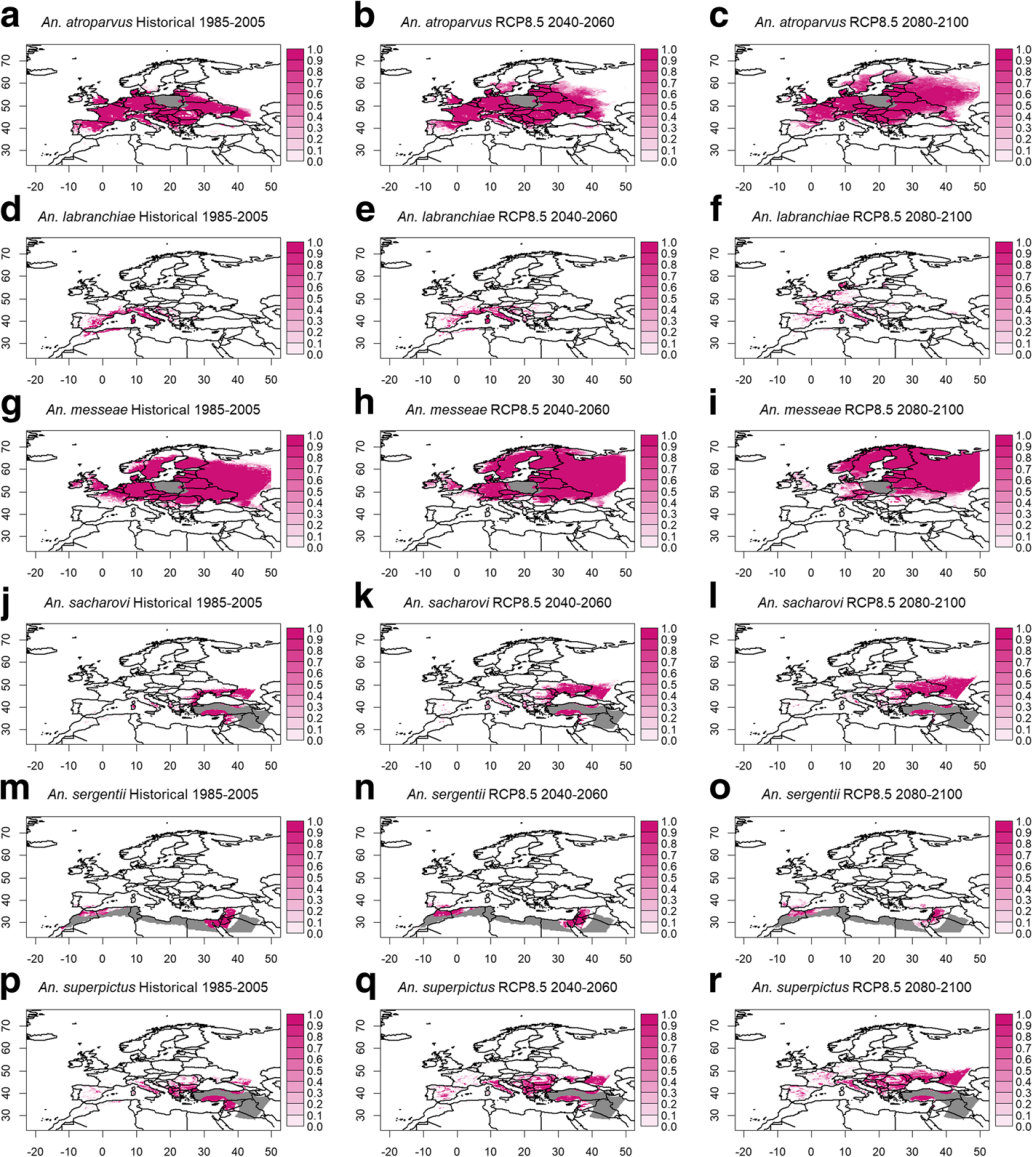
Modelled probabilities of vector occurrences. Shown are the results for the historical period 1985–2005, and the two scenario periods 2040–2060 and 2080–2100 under RCP8.5 scenario for *An. atroparvus* (**a-c**), *An. labranchiae* (**d-f**), *An. messeae* (**g-i**), *An. sacharovi* (**j-l**), *An. sergentii* (**m-o**), and *An. superpictus* (**p-r**). Shown is the ensemble mean from the two RCMs KNMI-RACMO22E and CLMcom-CCLM4-8-17. Grid boxes with vector presence in the observational period but no available observational climate data are marked in grey. Also, note the eastern boundary of the RCM domain at 45°E

Figure 1 shows the modelled probabilities of vector occurrences for the historical period 1985–2005, and the two scenario periods 2040–2060 and 2080–2100 under the RCP8.5 scenario for each vector. Shown is the mean value from the BRT predictions using bias-corrected temperature and precipitation of the two RCMs KNMI-RACMO22E and CLMcom-CCLM4-8-17 as new predictor data. Projections based on the RCP4.5 scenario yielded similar, although weaker tendencies of distribution changes.

Most noticeable is the northward spread of the vectors *An. atroparvus* and *An. messeae* until the end of the 21st century, with concurrent disappearance of *An. messeae* over the western parts of Europe. *Anopheles labranchiae*, *An. sacherovi* and *An. superpictus* also showed northward extensions, but often with lower occurrence probabilities in the newly emerging areas. In contrast, vector occurrences in the Mediterranean area were generally mostly declining. Most pronounced was the reduction of the occurrence area of *An. superpictus*, *An. sacharovi* and *An. sergentii* over the eastern Mediterranean area and North Africa under future climate conditions.

The distribution changes were strongly governed by the general increases of temperature. In particular, the strong temperature increases over north-eastern Europe and the Mediterranean area in spring and autumn played a role in this regard. Furthermore, changes of the precipitation pattern in summer, with increases over north-eastern Europe and strong decreases over the Mediterranean area as well as southern and western Europe, accounted for the changes, specifically for *An. messeae* and *An. sergentii*. Since both, and in particular *An. sergentii*, heavily rely on summer rainfall, their occurrence probabilities were noticeably reduced until the end of the 21st century.

### Potential malaria transmission stability in the 21st century

Potential malaria transmission stability during the 21st century was assessed based on the VSI. The spatial index includes the most important intrinsic properties of anopheline mosquito vectors of malaria that interact with climate to determine vectorial capacity. The index examines potential transmission stability, and thus the index includes “anophelism (with as well as) without malaria” [15].

**Fig. 2:**
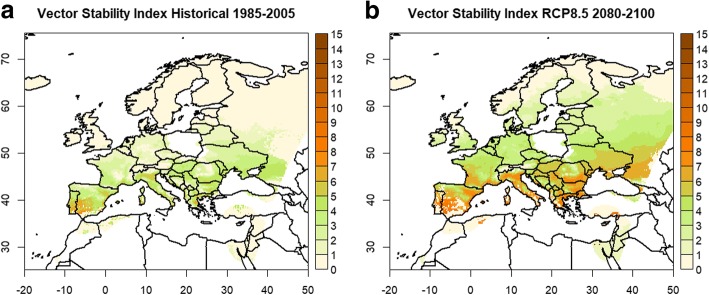
Vector Stability Index. Shown are the values for the historical period 1985–2005 (**a**) and for the scenario period 2080–2100 under the RCP8.5 scenario (**b**). White areas denote regions with no observational and/or RCM data

Figure 2 depicts the VSI for the historical period 1985–2005 as well as for the period 2080–2100 under the RCP8.5 scenario assumptions. RCM ensemble mean temperatures were used as climate data input for the calculation of the length of the extrinsic incubation period. Under historical climate conditions, there was in general a low force of transmission throughout Europe. Under RCP.8.5 conditions, large parts of the southern and south-eastern European area emerged as regions with a relatively high stability of potential malaria transmission. From the belt of high VSI values southwards towards North Africa as well as northwards towards Scandinavia, transmission stability declined. The decrease of malaria transmission stability to the south was mainly related to the projected rainfall reductions and the resulting decline of vector occurrences due to the drought-induced inhibition of the aquatic life-cycle of the vectors *An. sergentii* and also partly *An. labranchiae*. In contrast, the general northward decline from the belt of high stability points to the limitation of malaria transmission by temperature. The values of the VSI in central and northern Europe are controlled primarily by *An. atroparvus*, in northern Scandinavia by *An. messeae*. The temperature increases until the end of the 21st century suffice to have the vectors spread throughout Europe. However, the climatic changes only impacted the transmission stability in southern and south-eastern Europe, whereas the length of the extrinsic incubation period was still temperature-limited over northern Europe.

## Discussion

The statistical models showed that occurrences of *An. atroparvus*, *An. labranchiae*, *An. messeae*, *An. sacharovi*, *An. sergentii* and *An. superpictus* are highly related to climate. This confirms the high climate sensitivity of *Anopheles* vectors as already identified in previous studies, e.g. [5, 12]. In this regard, temperature in the transitional seasons as well as rainfall during summer are of particular importance. The results point to the impact of climate on specific parts of the vectors life-cycle - temperature mainly influences the active and hibernation periods of adult mosquitoes, whereas rainfall is particularly important for the aquatic life stages. Precipitation was mostly positively connected with *Anopheles* occurrences. However, very high monthly precipitation amounts can also have a reverse impact on occurrences. *Anopheles messeae* and *An. sergentii* prefer freshwater sites with very slow flowing or stagnant water [28], so that a prevention of breeding sites by high rainfall amounts can occur. *Anopheles sacharovi* is described as a highly adaptive species, developing in all kinds of brackish as well as fresh water habitats [28]. However, the species is sensible to organic pollutants [29]. Thus, it can be speculated that intense rainfall events may import pollutants into its habitat yielding a reduced species occurrence.

Despite the finding that climatic predictors perform much better in the BRTs compared to land use information, it should be investigated how future land use changes may change vector occurrences. In the past, drainage of wetlands and new farming techniques substantially contributed to the eradication of malaria in Europe [4, 28]. In contrast, ongoing land use changes such as crop irrigation and urbanization may create new breeding sites [30]. In the present study *An. atroparvus* and *An. labranchiae* occurrences were found to be positively related to cropland, whereas *An. labranchiae* and *An. superpictus* occurrences were negatively related to shrubland. It remains to be investigated how dynamic land use changes will alter the vector distributions in the future. A promising approach represents the Flagship Pilot Study “LUCAS” (Land Use & Climate Across Scales) for Europe, as a EURO-CORDEX & LUCID initiative, giving the prospect to consider consistently land use and climate changes in future studies of vector distribution.

The present study highlights that distinct changes in the distributions of the dominant vectors of human malaria are to be expected under the constraints of future climate change. Temperature and precipitation changes will lead to a northward spread of the occurrences of *Anopheles* vectors. This result is in general agreement with the findings from the global-scale, multi-model malaria assessment of Caminade et al. [6]. However, RCMs provide more detailed spatial patterns compared to GCMs, which allows for an improved analysis of the relationships of vector distribution and transmission stability with climatic changes. Thus, the high-resolution projections of the present study showed that for some Mediterranean areas occurrence probabilities may decline, mainly due to projected rainfall decreases.

Furthermore, the modelled expansions of vector distributions in the future do not automatically imply a concurrent increase of the potential malaria transmission stability. Transmission stability was only increased in areas where the climatic changes favor vector occurrences as well as yield enough temperature rise to significantly impact the vectorial capacity. As a consequence of this, the VSI showed the highest increases between historical and future periods for the southern and south-eastern European areas. *Anopheles atroparvus* is the dominant vector in large parts of Europe under present as well as future climate conditions. In addition, it has the highest human-biting proportion and daily survival rate of the European vectors [15]. The increases of the duration of the transmission season and of the extrinsic incubation period assigns this vector an important role with respect to changes of the potential transmission stability. Countries affected by an increased malaria risk comprised for instance Spain, southern France, Italy, Greece, the eastern European countries Bulgaria, Romania, Macedonia and Serbia as well as southern Ukraine and Russia (Fig. 2). For Turkey no information was available due to the widespread lack of reliable observational climate data.

This study is one of the first high-resolution assessment of the impact of future climate change on vector distributions and potential transmission stability in the European and Mediterranean area using state-of-the-art climate scenarios. Further improvements may be possible through the consideration of a more diverse influence of temperature on the transmission intensity of malaria. For instance, Shapiro et al. [31] experimentally showed by means of *An. stephensi* and *P. falciparum* that there are complex relationships of temperature with adult mosquito longevity, human-biting rate, the developmental period of the parasite within the mosquito, and the proportion of mosquitoes that become infectious. Paajmans et al. [32] illustrated that the extrinsic incubation period of *P. falciparum* is modified by including the diurnal temperature range and day length in comparison to estimates based on mean temperature values only. While there is a high climate model agreement with respect to mean temperature and precipitation changes, the use of extremes and further explanatory variables, like e.g. wind speed or evapotranspiration will require a careful evaluation of the projection uncertainties related to these variables. Appropriate downscaling techniques have to be applied in this context, which involves the availability of reliable observational data as reference as well as specific methods, which preserve the physical consistency between variables and adequately adjust the extreme parts of the distributions [33].

Furthermore, important non-climatic factors such as population growth and urbanization, migration changes, and economic development should be considered for future risk assessments. Malaria declined rapidly in Europe during the 20th century due to the implementation of national elimination programs, involving for instance draining of wetlands, insecticide spraying, and improvements of health infrastructures [4]. Yet a high number of imported malaria cases from endemic to non-endemic countries is reported, e.g. 2169 cases per year in the period 2005–2015 for France, 637 cases for Italy, 374 cases for Spain [34]. In addition, the local reappearance of malaria in some parts of southern Europe is observed in recent years [3]. Migration and economic hardship are considered as critical variables with respect to the vulnerability of a region [4]. Moreover, with respect to ongoing urbanization, novel breeding sites become available. For instance, special attention has recently been given to *An. plumbeus*, which exploits man-made breeding sites, is able to transmit *P. falciparum*, and has a high human-biting proportion [35]. Thus, further investigations should also consider this vector. In addition, very high-resolution climate and land-use modeling, which resolves the sub-urban scale, should be used for risk assessment.

## Conclusions

Potential malaria transmission stability is increased in areas where the climatic changes favor vector occurrences as well as significantly impact the vectorial capacity. As a result, vector stability shows the highest increases between historical and future periods for the southern and south-eastern European areas. *Anopheles atroparvus*, the dominant vector in large parts of Europe, might play an important role with respect to changes of the potential transmission stability. Risk assessments of malaria in view of climate change as well as of other factors like land use changes and ongoing urbanization are of particular importance on the local scale. The present contribution adds to the current research by providing high-resolution projections of climate-induced changes in Europe and the Mediterranean area.

## Additional file

Additional file 1: **Figure S1.** Distribution maps of the dominant *Anopheles* vectors in Europe and the Mediterranean area: *An. atroparvus* (a); *An. labranchiae* (b); *An. messeae* (c); *An. sacharovi* (d); *An. sergentii* (e); and *An. superpictus* (f). Data taken from Sinka et al. [5]. **Figure S2.** Modelled probabilities of vector occurrences in the observational period 1985-2009: *An. atroparvus* (a); *An. labranchiae* (b); *An. messeae* (c); *An. sacharovi* (d); *An. sergentii* (e); and *An. superpictus* (f). Grid boxes with vector presence but no available climate data are marked in grey. **Table S1.** Performance of Empirical Quantile Mapping of daily RCM output. *Abbreviations*: RMSE: root mean square error (precipitation in mm/day, temperature in K); Raw: raw RCM output; QM: Quantile Mapping results. Shown is for each month the mean performance over the two validation periods. **Table S2.** Performance of Boosted Regression Trees. Shown are the evaluation statistics based on the model development independent data. (DOCX 666 kb)

## Abbreviations

BRT: Boosted regression trees
GCM: General circulation model
RCM: Regional climate model
VSI: Vector stability index
