# Supplementary material for: Distribution of Anopheles vectors and potential malaria transmission stability in Europe and the Mediterranean area under future climate change
Source: Parasit Vectors. 2019 Jan 8;12:18. doi: 10.1186/s13071-018-3278-6 (PMC6325871; doi:10.1186/s13071-018-3278-6)
Supplement: Supplementary file 1 — Figure S1. Distribution maps of the dominant Anopheles vectors in Europe and the Mediterranean area: An. atroparvus (a); An. labranchiae (b); An. messeae (c); An. sacharovi (d); An. sergentii (e); and An. superpictus (f). Data taken from Sinka et al. [5]. Figure S2. Modelled probabilities of vector occurrences in the observational period 1985-2009: An. atroparvus (a); An. labranchiae (b); An. messeae (c); An. sacharovi (d); An. sergentii (e); and An. superpictus (f). Grid boxes with vector presence but no available climate data are marked in grey. Table S1. Performance of Empirical Quantile Mapping of daily RCM output. Abbreviations: RMSE: root mean square error (precipitation in mm/day, temperature in K); Raw: raw RCM output; QM: Quantile Mapping results. Shown is for each month the mean performance over the two validation periods. Table S2. Performance of Boosted Regression Trees. Shown are the evaluation statistics based on the model development independent data. (DOCX 666 kb) [file 13071_2018_3278_MOESM1_ESM.docx]

**Distribution of *Anopheles* vectors and potential malaria transmission stability in Europe and the Mediterranean area under future climate change**

Elke Hertig^1^*

^1^Institute of Geography, University of Augsburg, Alter Postweg 118, 86135 Augsburg, Germany

*Correspondence: elke.hertig@geo.uni-augsburg.de


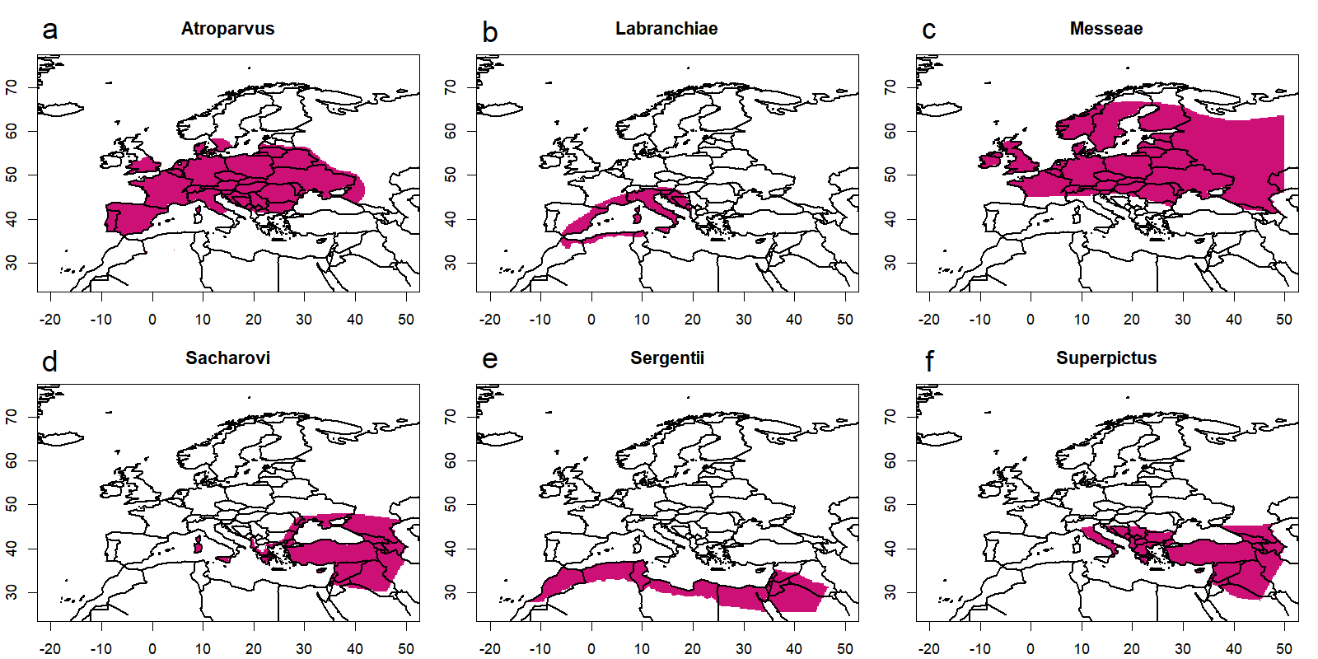


Figure S1. Distribution maps of the dominant *Anopheles* vectors in Europe and the Mediterranean area: *An. atroparvus* (a), *An. labranchiae* (b), *An. messeae* (c), *An. sacharovi* (d), *An. sergentii* (e), and *An. superpictus* (f). Data taken from Sinka et al. (2010) [1].


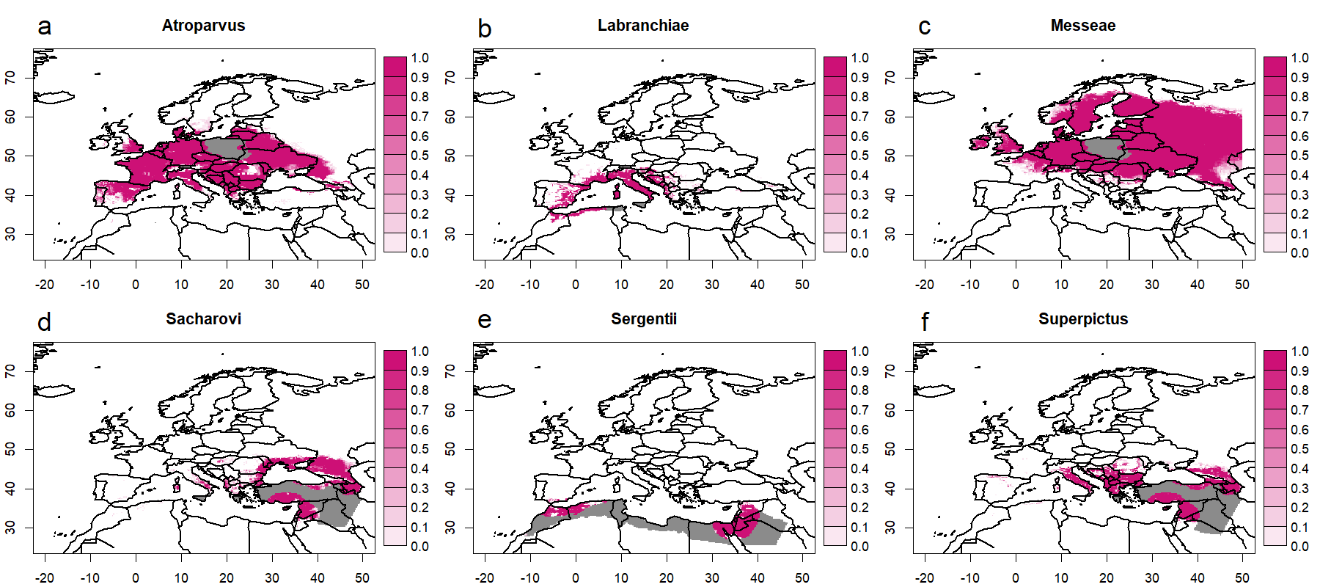


Figure S2. Modelled probabilities of vector occurrences in the observational period 1985-2009 modelled with Boosted Regression Trees: *An. atroparvus* (a), *An. labranchiae* (b), *An. messeae* (c), *An. sacharovi* (d), *An. sergentii* (e), and *An. superpictus* (f). Grid boxes with vector presence but no available climate data are marked in grey.

Table S1. Performance of Empirical Quantile Mapping of daily RCM output. RMSE: root mean square error (precipitation in mm/day, temperature in K). Raw: raw RCM output, QM: Quantile Mapping results. Shown is for each month the mean performance over the two validation periods.

| **ICHEC-EC-EARTH**  **KNMI-RACMO22E** |  |  |  |  |  |  |  |  |  |  |  |  |
| --- | --- | --- | --- | --- | --- | --- | --- | --- | --- | --- | --- | --- |
|  | **Jan** | **Feb** | **Mar** | **Apr** | **May** | **Jun** | **Jul** | **Aug** | **Sep** | **Oct** | **Nov** | **Dec** |
| **Precipitation** |  |  |  |  |  |  |  |  |  |  |  |  |
| **RMSE raw** | 0.85 | 0.66 | 0.63 | 0.55 | 0.54 | 0.49 | 0.48 | 0.55 | 0.52 | 0.57 | 0.62 | 0.77 |
| **RMSE QM** | 0.55 | 0.52 | 0.54 | 0.35 | 0.32 | 0.46 | 0.36 | 0.36 | 0.39 | 0.47 | 0.41 | 0.48 |
| **Temperature (mean)** |  |  |  |  |  |  |  |  |  |  |  |  |
| **RMSE raw** | 2.35 | 2.18 | 2.84 | 3.39 | 3.07 | 2.66 | 2.25 | 1.98 | 2.14 | 2.19 | 2.56 | 2.26 |
| **RMSE QM** | 1.32 | 1.03 | 1.01 | 1.09 | 0.99 | 1.16 | 0.56 | 0.80 | 0.84 | 0.73 | 2.08 | 2.10 |
| **Temperature (min)** |  |  |  |  |  |  |  |  |  |  |  |  |
| **RMSE raw** | 2.56 | 2.53 | 3.37 | 4.30 | 3.67 | 2.90 | 2.41 | 2.13 | 2.26 | 2.43 | 2.80 | 2.45 |
| **RMSE QM** | 1.42 | 1.16 | 1.08 | 1.20 | 0.96 | 0.99 | 0.66 | 0.78 | 0.91 | 0.78 | 2.20 | 2.22 |
| **Temperature (max)** |  |  |  |  |  |  |  |  |  |  |  |  |
| **RMSE raw** | 2.30 | 2.16 | 2.91 | 3.58 | 3.73 | 3.64 | 3.25 | 2.73 | 2.61 | 2.22 | 2.36 | 2.05 |
| **RMSE QM** | 1.22 | 0.87 | 1.16 | 1.09 | 0.94 | 1.26 | 0.65 | 0.94 | 0.89 | 0.76 | 1.99 | 1.94 |
|  |  |  |  |  |  |  |  |  |  |  |  |  |
| **MPI-M-MPI-ESM-LR CLMcom-CCLM4-8-17** |  |  |  |  |  |  |  |  |  |  |  |  |
|  | **Jan** | **Feb** | **Mar** | **Apr** | **May** | **Jun** | **Jul** | **Aug** | **Sep** | **Oct** | **Nov** | **Dec** |
| **Precipitation** |  |  |  |  |  |  |  |  |  |  |  |  |
| **RMSE raw** | 0.96 | 0.88 | 0.91 | 0.87 | 0.99 | 0.84 | 0.76 | 0.73 | 0.72 | 0.80 | 0.85 | 0.85 |
| **RMSE QM** | 0.66 | 0.59 | 0.35 | 0.35 | 0.42 | 0.51 | 0.39 | 0.39 | 0.40 | 0.53 | 0.61 | 0.46 |
| **Temperature (mean)** |  |  |  |  |  |  |  |  |  |  |  |  |
| **RMSE raw** | 1.38 | 1.41 | 1.23 | 1.06 | 1.35 | 1.81 | 2.03 | 1.79 | 1.21 | 1.16 | 1.21 | 1.35 |
| **RMSE QM** | 1.09 | 1.18 | 0.78 | 0.60 | 0.82 | 0.53 | 0.55 | 0.69 | 0.41 | 0.54 | 1.03 | 1.35 |
| **Temperature (min)** |  |  |  |  |  |  |  |  |  |  |  |  |
| **RMSE raw** | 1.80 | 1.60 | 1.58 | 1.33 | 1.13 | 1.09 | 1.23 | 1.14 | 1.28 | 1.16 | 1.32 | 1.63 |
| **RMSE QM** | 1.18 | 1.42 | 0.88 | 0.58 | 0.67 | 0.46 | 0.53 | 0.59 | 0.50 | 0.62 | 1.08 | 1.53 |
| **Temperature (max)** |  |  |  |  |  |  |  |  |  |  |  |  |
| **RMSE raw** | 1.78 | 2.11 | 2.04 | 2.12 | 2.63 | 3.48 | 3.73 | 3.47 | 2.31 | 2.05 | 1.72 | 1.71 |
| **RMSE QM** | 1.07 | 1.06 | 0.83 | 0.77 | 0.98 | 0.73 | 0.68 | 0.95 | 0.53 | 0.65 | 1.00 | 1.20 |

Table S2. Performance of Boosted Regression Trees. Shown are the evaluation statistics on from the model development independent data.

|  | **Deviance** | **Correlation** | **Discrimination** | **Kappa** |
| --- | --- | --- | --- | --- |
| ***An. atroparvus*** | **0.61** | **0.85** | **0.97** | **0.79** |
| ***An. labranchiae*** | **0.24** | **0.72** | **0.97** | **0.66** |
| ***An. messeae*** | **0.73** | **0.82** | **0.98** | **0.75** |
| ***An. sacharovi*** | **0.31** | **0.83** | **0.98** | **0.80** |
| ***An. sergentii*** | **0.11** | **0.85** | **0.99** | **0.81** |
| ***An. superpictus*** | **0.28** | **0.85** | **0.98** | **0.82** |

**References**

1. Sinka ME, Bangs MJ, Manguin S, Coetzee M, Mbogo CM, Hemingway J, et al. The dominant *Anopheles* vectors of human malaria in Africa, Europe and the Middle East: occurrence data, distribution maps and bionomic précis. Parasit Vectors. 2010;3:117.
